# Crystal Structures of Multidrug Binding Protein TtgR in Complex with Antibiotics and Plant Antimicrobials

**DOI:** 10.1016/j.jmb.2007.03.062

**Published:** 2007-06-08

**Authors:** Yilmaz Alguel, Cuixiang Meng, Wilson Terán, Tino Krell, Juan L. Ramos, María-Trinidad Gallegos, Xiaodong Zhang

**Affiliations:** 1 Centre for Structural Biology and Division of Molecular Biosciences, Imperial College London, London, SW7 2AZ, UK; 2 Departamento de Bioquímica, Biología Molecular y Celular de Plantas Estación Experimental del Zaidín Profesor Albareda, 1 E18008-Granada, Spain

**Keywords:** crystal structure, multidrug binding, antibiotic resistance, ITC, protein–ligand interaction

## Abstract

Antibiotic resistance is a widely spread phenomenon. One major mechanism that underlies antibiotic resistance in bacteria is the active extrusion of toxic compounds through the membrane-bound efflux pumps that are often regulated at the transcriptional level. TtgR represses the transcription of TtgABC, a key efflux pump in *Pseudomonas putida*, which is highly resistant to antibiotics, solvents and toxic plant secondary products. Previously we showed that TtgR is the only reported repressor that binds to different classes of natural antimicrobial compounds, which are also extruded by the efflux pump. We report here five high-resolution crystal structures of TtgR from the solvent-tolerant strain DOT-T1E, including TtgR in complex with common antibiotics and plant secondary metabolites. We provide structural basis for the unique ligand binding properties of TtgR. We identify two distinct and overlapping ligand binding sites; the first one is broader and consists of mainly hydrophobic residues, whereas the second one is deeper and contains more polar residues including Arg176, a unique residue present in the DOT-T1E strain but not in other *Pseudomonas* strains. Phloretin, a plant antimicrobial, can bind to both binding sites with distinct binding affinities and stoichiometries. Results on ligand binding properties of native and mutant TtgR proteins using isothermal titration calorimetry confirm the binding affinities and stoichiometries, and suggest a potential positive cooperativity between the two binding sites. The importance of Arg176 in phloretin binding was further confirmed by the reduced ability of phloretin in releasing the mutant TtgR from bound DNA compared to the native protein. The results presented here highlight the importance and versatility of regulatory systems in bacterial antibiotic resistance and open up new avenues for novel antimicrobial development.

## Introduction

Microorganisms are exposed to naturally occurring deleterious chemicals in the environment, like natural antibiotics produced by bacteria, fungi and plants, or detergents such as bile salts, present in the intestinal tract of higher animals. Human activity has led to the emergence of a great diversity of noxious chemicals. Furthermore, some chemicals, such as semi-synthetic antibiotics or biocides, have been specifically developed to act as antimicrobial agents. However, bacteria display resistance to the action of these toxic compounds through intrinsic long-standing mechanisms that protect the bacterial cells from continuous exposure to the toxic compounds. The extrusion of toxic compounds by efflux pumps is a major cell protective mechanism as it provides an active outward transport of deleterious compounds from the cytoplasmic membrane into the external medium and reduces the concentration of antimicrobials in the membranes and/or the cytoplasm to sub-toxic levels.^1,2^ A number of studies have shown that the expression of multidrug resistance (MDR)^1^ efflux pumps is controlled by transcriptional regulators with the same multidrug recognition properties, such as BmrR of *Bacillus subtilis*,^3^ EmrR of *Escherichia coli*,^4^ QacR of *Staphylococcus aureus*^5^ and TtgR of *Pseudomonas putida* DOT-T1E.^6,7^ Studies on these transcriptional regulators have shown that they act by directly binding to a wide range of similar toxic compounds to that exported by the membrane proteins whose expression they control, thereby facilitating the induction of efflux pump genes in response to the presence of diverse toxic substances.

*P. putida* is a non-pathogenic bacterium present in water and soil that is able to colonize plant roots and seeds.^8,9^ The DOT-T1E strain was isolated for its particularly high resistance to toxic organic solvents.^10^ Three efflux pumps, TtgABC, TtgDEF and TtgGHI were found to be essential for this resistance.^11^ Divergently transcribed from *ttgABC operon* is *ttgR*, which encodes the corresponding regulator. TtgR is a multidrug binding repressor that negatively controls the transcription of the *ttgABC* operon as well as its own expression. TtgR belongs to the TetR family of proteins, which are characterized by a conserved DNA binding domain but variable ligand binding domain. The TtgR-TtgABC system has been shown to recognize and extrude compounds that belong to different functional classes including antibiotics, flavonoids and organic solvents^12–14^; all of these compounds are toxic to the bacterial cell. Flavonoids are present in the soil as they are released from plant roots as a defensive mechanism. Quercetin, a plant secondary metabolite, was previously shown to possess antibacterial activity^15,16^ through the inhibition of bacterial DNA gyrases.^17^ However, the exposure of *P. putida* to organic solvents is mainly due to human activity. The toxicity of aromatic organic solvents is founded in its capacity to dissolve into the membrane leading to its damage.

The various compounds found to be relevant for the TtgR-TtgABC system differ in structure and toxic mechanism. We have previously shown that these compounds act directly on TtgR, resulting in increased tolerance of *P. putida* DOT-T1E to antimicrobials.^6,13^ Here we provide the structural basis for the binding of different types of natural ligands to TtgR, including antibiotics (chloramphenicol and tetracycline), and plant antimicrobials (phloretin, quercetin and naringenin). Together these structures presented here unveil the mechanism employed by TtgR to recognize and bind to a wide range of aromatic compounds.

## Results

### Overall TtgR structure

**Figure 1:**
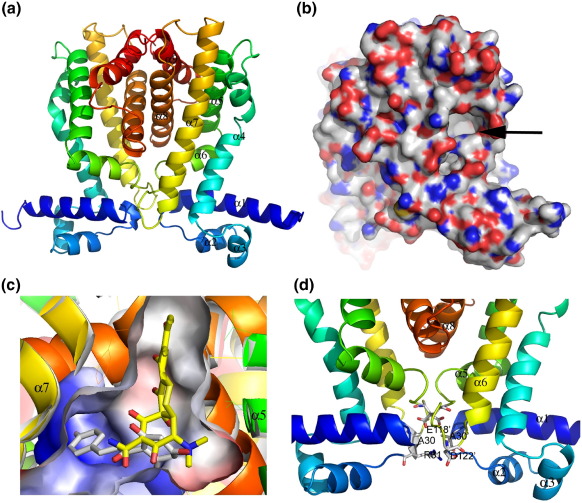
TtgR structures. (a) Ribbon diagram of TtgR dimer with helices labelled from N to C termini. Blue, N-terminal; red, C-terminal. (b) Molecular surface of TtgR dimer calculated using PyMol clearly shows the cleft (arrow). Red, oxygen; blue, nitrogen; white, carbon. (c) Ligand binding pocket of TtgR calculated using PyMol. (d) Closed-up view of TtgR dimer interface, contributed by both the ligand binding domain and the DNA binding domain.

**Figure 2:**
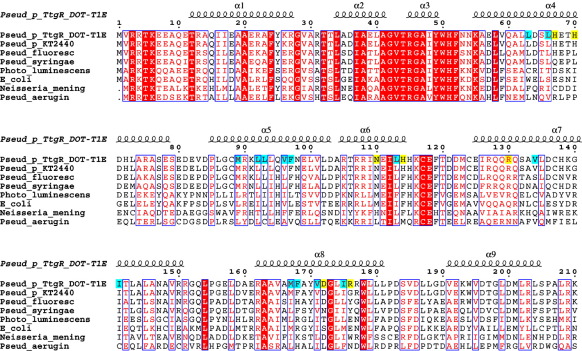
Sequence alignment of TtgR from *P. putida* DOT-T1E with its close homologues. Residues involved in effector binding are shaded in cyan for hydrophobic or yellow for polar residues. Secondary structure elements are labelled. Completely conserved residues are shaded in red while highly conserved residues are coloured in red.

Since TtgR crystals diffract poorly in the absence of its ligands, we therefore focus here on TtgR structures in complex with different ligands. The structure of TtgR in complex with chloramphenicol was determined using selenomethionine substituted crystals and multiple-wavelength anomalous diffraction (MAD) methods and all subsequent structures were determined by molecular replacement method using the TtgR/chloramphenicol structure without the ligand as a starting model. A TtgR monomer consists of nine alpha helices forming two distinct domains (Figure 1(a)). The DNA binding domain consists of α1–α3 (residues 1–53) while the ligand-binding domain consists of α4–α9 (residues 54–210), with an angle of approximately 80° between the two domains. The most unusual feature of the TtgR structure is that there is a 65° inward bending in the middle of α4 (residues 55–78, Figure 1(a)) compared to other family members. This bend is at His70 (with a Phi Psi angle of ∼(− 100°, 10°) compared to ∼(− 60°, − 50°) for a typical α-helix) and results in a relatively large portal formed between α4 and α7 (residues 125–150, Figure 1(a)). This bend also closes the channel at the top of the ligand binding domain where the C-terminal half of α4 (residues 71–78), α5 (residues 85–103), and α7 (residues 125–150) form a helical bundle. α4 is not involved in crystal contact or dimerization, hence this bend is a distinct and native feature of TtgR. The molecular surface of the TtgR dimer reveals a cleft around the bend, ideal for ligand binding (Figure 1(b)). Closer examination of the cleft and the ligands (see next section) reveals a large triangular ligand-binding pocket, which is largely hydrophobic (Figure 1(c)). One TtgR dimer is found within the crystallographic asymmetric unit and no crystallographic symmetry restraint is applied. Interestingly, the individual monomers are almost identical (rmsd C^α^ = 0.5 Å) although some side-chain differences are observed within the ligand binding pocket. α8 (residues 162–180), α9 (residues 191–205), the loop connecting α6 and α7 (specifically residues Glu118, Asp122), as well as the loop connecting α1 and α2 (specifically residues Ala30, Arg31) contribute to the dimer interface (Figure 1(d)). All TetR family members bind to DNA as a homodimer utilizing the helix-turn-helix (HTH) motif.^18^ The distance between the DNA recognition helices (α3, residues 45 to 49) is 42 Å, significantly larger than the 34 Å major groove distance of *B*-DNA. Sequence alignment with TtgR close homologues confirms the highly conserved nature of the DNA binding domain with a relatively diverse ligand-binding domain (Figure 2).

### TtgR in complex with antibiotics

**Figure 3:**
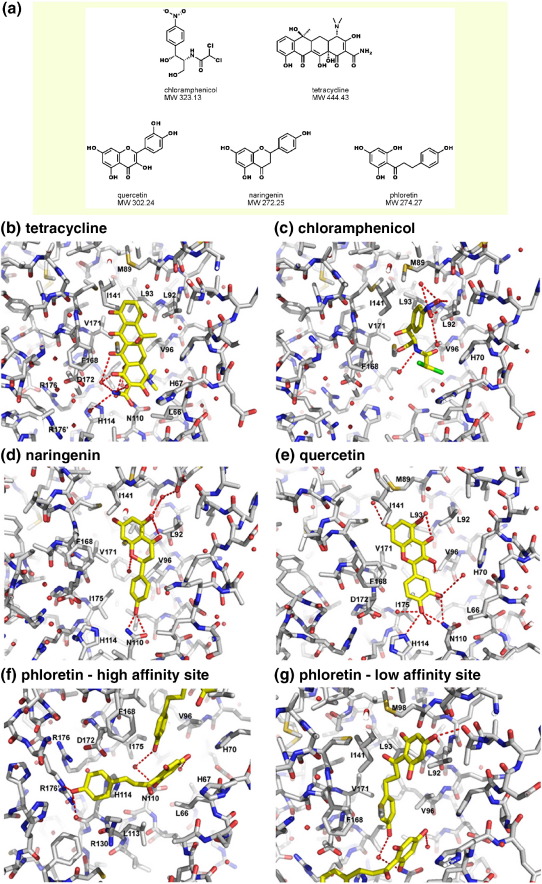
Detailed effector binding and interactions. (a) Chemical structures of the effector molecules characterized in this study. (b) Tetracycline binding. (c) Chloramphenicol binding. (d) Naringenin binding. (e) Quercetin binding. (f) High affinity phloretin binding. (g) Low affinity phloretin binding. Effector molecules are displayed as sticks. Residues contributing to the binding sites are labelled and colour-coded according to atomic properties. O, red; N, blue; C, white for protein or yellow for ligand; S, orange; Cl, green. Interactions between ligands and TtgR residues as well as water molecules (red spheres) are represented by broken lines. Ligand binding sites were analysed using PyMol with a 3.6 Å cut off for hydrogen bonds.

TtgR can recognize a wide range of molecules including a number of antibiotics, flavonoids, as well as aromatic solvents. The ligands differ in size (with molecular mass ranging from 272 to 444 Da) and shape, as well as their charge distributions (Figure 3(a)). The only common feature of the ligands is the presence of at least one aromatic ring.

**Supplementary Figure S1:**
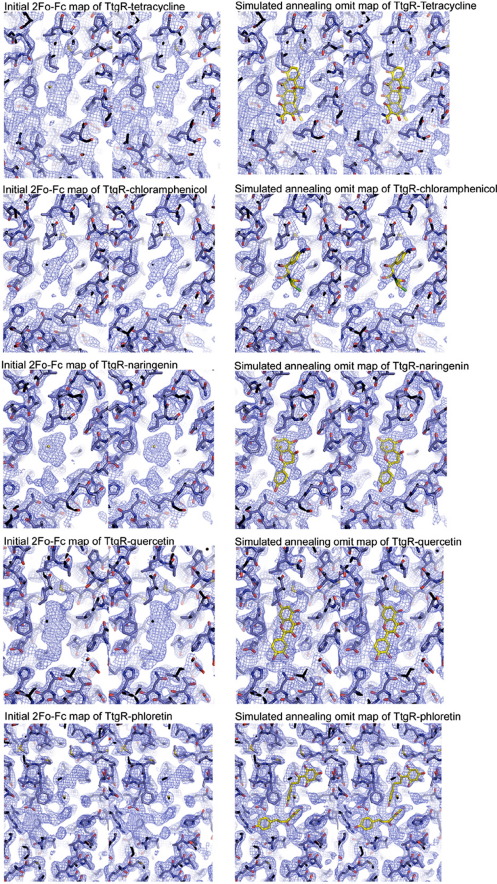
Stereo images of initial and final simulated annealing omit 2Fo-Fc electron density maps of TtgR-ligand structures showing the TtgR effector binding pocket contoured at 1σ level. Effector molecules are displayed as stick models and colored in yellow. Note the two phloretin molecules within the same pocket. The horizontal one has better defined electron density than the vertical one.

To characterize the structural basis for antibiotic binding to TtgR, we determined the crystal structures of TtgR in complex with tetracycline and chloramphenicol, both at 2.7 Å resolution. The initial 2*F*_o_–*F*_c_ map obtained using phases from the TtgR model in the absence of ligands showed clear ligand density (Supplementary Data Figure 1). Almost all secondary structural elements in the ligand binding domain are involved in tetracycline binding. More specifically, the ligand binding pocket is formed by residues from α4 (residues 55–78), α5 (residues 85–103), α6 (105–114), α7 (125–150), and α8 (162–181) (Figures 1(a) and 3(b)). The ligand binding pocket is relatively large, with a volume of ∼ 1500 Å^3^. Looking from the portal formed by α4 (55–78) and α7 (125–150) (Figures 1(a)–(c) and 3(b)), the ligand binding pocket is an asymmetric pyramid with α7 (125–150) and α8 (162–181) forming one side of the wall, while the top halves of α4 (71–78) and α5 (85–103) contribute to the other sides. The lower half of α4 (55–69) together with α6 (105–114) forms the bottom wall. The top of the pocket (far end from the DNA binding domain) contains hydrophobic residues Ile141, Met89, and Met167, just as the side walls do with Leu92, Leu93, Val96, Phe168, and Val171. The bottom of the pocket however consists of polar residues including Asn110, His114, and Asp172 (Figure 3(b) and (c)). This is also reflected by the charge distribution within the pocket (Figure 1(c)). The tetracycline molecule lies almost parallel to the TtgR dimer axis with the dimethylamino group located near the widest point of the pocket, where α4 bends (Figure 1(a) and (c)). Not surprisingly, the cyclic rings are surrounded by the side wall hydrophobic residues including Leu92, Leu93, Val96 (α4), Ile141 (α7), and Phe168 (α8) that constitute the side wall (Figures 2 and 3(b)). The hydroxyl groups of tetracycline interact with the main chain N atoms and the side-chains of Asp172. Asp172 also interacts with a number of hydroxyl groups as well as the amino group through water molecules. Interestingly, Asn110 is ideally positioned to coordinate the interaction with both the dimethylamino and amino groups.

The structure of TtgR in complex with chloramphenicol was also determined at 2.7 Å resolution. The TtgR structure is almost identical to that of TtgR in complex with tetracycline. Chloramphenicol is situated similarly to tetracycline and utilizes the general hydrophobic environment of the side walls with little specific interactions (Figure 3(c)).

### TtgR in complex with flavonoids

Several flavonoids have antimicrobial properties, and indeed, their ability to bind to TtgR and induce the expression of the corresponding TtgABC efflux pump was recently demonstrated.^13^ We obtained structures of TtgR in complex with three flavonoids: naringenin, quercetin, and phloretin and these structures provide a detailed understanding of the recognition of flavonoids by TtgR.

#### TtgR in complex with quercetin and naringenin

Quercetin and naringenin have similar structures consisting of a chromenone ring and a hydroxyphenyl ring (Figure 3(a)). The two flavonoids differ by additional hydroxyl groups in quercetin. Both effectors bind at similar locations to the hydrophobic binding site as tetracycline and chloramphenicol. The chromenone rings are located near the top of the binding pocket and are surrounded by Leu92, Leu93 (α5), Ile141 (α7) and Phe168 (α8) while the hydroxyphenyl ring is surrounded by Leu66 (α4) and Val96 (α5), V171, and I175 (α8). A number of water molecules also contribute to the interactions with the chromenone ring. In the TtgR-naringenin complex, the density for the hydroxyphenyl ring is poor, reflecting the flexibility of the molecular structure, even though the hydroxyphenyl ring is located at the bottom of the pocket and can form hydrogen bond with side-chains of Asn110 (Supplementary Data Figure 1 and Figure 3(d)). The electron density for quercetin is better defined (Supplementary Data Figure 1). The extra hydroxyl groups in the chromenone ring interact with additional water molecules. The hydroxyphenyl group is well defined, forming interactions with side-chains of Asn110 and His114 (Figure 3(e)). We propose that the increased binding affinity of quercetin to TtgR (compared to naringenin) is due to the additional interactions formed between the extra hydroxyl groups of quercetin (compared to naringenin) and TtgR as well as water molecules.^13^ The increased affinity is also reflected in the much better defined electron density for quercetin compared to naringenin. The quercetin and naringenin binding sites involve exclusively residues of a single monomer, and it is thus not surprising that one dimer of TtgR can bind to two ligand molecules as observed in the structures.

It is worthwhile to note that Asn110 at the bottom of the ligand binding pocket interacts with both positive (as in tetracycline) and negative (as in narigenin and quercetin) charges using either OD1 or ND2 of its side-chains. Asn110 therefore plays an important role in the ability of TtgR to bind to both positively and negatively charged ligands.

#### TtgR in complex with phloretin

Phloretin contains a 2,4,6-trihydroxyphenyl ring connected to a 4-hydroxyphenyl ring. Contoured at 1σ level, the 2*F*_o_–*F*_c_ electron density observed in one of the binding pockets can easily accommodate two phloretin molecules, one occupying a similar location like that of tetracycline and quercetin, while the second one lies horizontally at the bottom of the binding pocket (Supplementary Data Figure 1). The density for the horizontal molecule is better defined than the vertical one, possibly indicating different affinities and occupancies. The horizontal molecule, which lies at the bottom and is buried deeply in the binding pocket (Figure 3(f)), makes multiple interactions between its hydroxyl groups and surrounding positively charged residues including His114, Arg130, and Arg176′ (where prime indicates the other monomer of the dimer) as well as a number of water molecules (Figure 3(f)). The trihydroxyphenyl also interacts with Asn110. A number of hydrophobic residues constitute the rest of the binding site including Leu66, Leu113, Val134, and Phe168. Interestingly, in the other monomer, only weak electron density is accountable for the vertical binding site, similar to the locations of tetracycline and other ligands. We therefore term the horizontal binding site to be the high affinity specific site and the vertical binding site to be the low affinity general binding site. Detailed comparison between the two TtgR monomers suggests that utilizing Arg176′ of the adjacent monomer in phloretin binding partially blocks the phloretin binding site in the adjacent monomer. Our observation suggests that only one phloretin molecule can bind to the specific site per TtgR dimer while additional phloretin molecules could bind to the general binding site, one in each TtgR monomer.

**Supplementary Figure S2:**
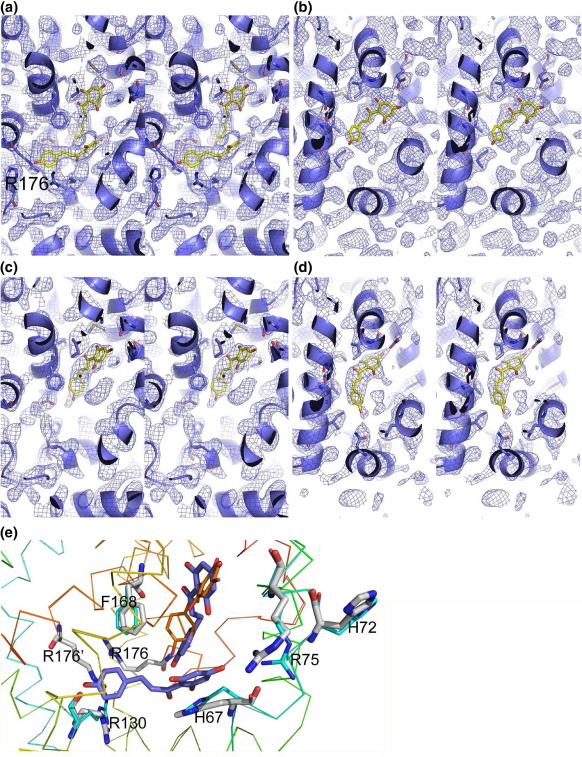
Stereo images of initial 2Fo-Fc maps showing density of phloretin binding to TtgR contoured at 1σ level. a) Two phloretin molecules are fitted into monomer A in wild type TtgR. b) One phloretin molecule is fitted in the adjacent monomer B. c) Only one phloretin molecule is observed in monomer A in TtgR^R176G^ crystals. d) One phloretin molecule is observed in monomer B in TtgR^R176G^ crystals. Phloretin molecules are shown as sticks. Arg176 is labeled. Color scheme: yellow–carbon atoms in phloretin, red–oxygen atoms, blue- nitrogen atoms. e) Structural superposition of TtgR (thick sticks) and TtgR^R176G^ (thin sticks). In TtgR structure, two phloretin molecules are present in one binding pocket, one molecule lying horizontally while the second one vertically. In TtgR^R176G^ structure, only one phloretin molecule is present lying in the vertical location.

**Figure 4:**
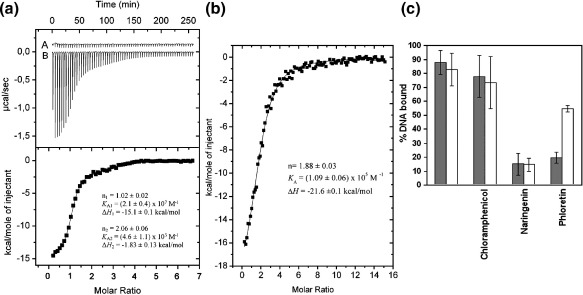
Isothermal titration calorimetry and EMSA studies of effector binding to native and mutant TtgR. (a) ITC measurement of phloretin binding to native TtgR. Upper panel: Heat changes for the injection of 1.6 μl aliquots of 2 mM phloretin into buffer (A) and into 22 μM dimeric TtgR (B). For clarity data were off-set on the *y*-axis. Lower panel: Integrated and dilution corrected peak areas of the raw data. Data were fitted with the “two-independent binding sites” model of ORIGIN (Microcal). (b) ITC measurement of phloretin binding to TtgR^R176G^. (c) EMSA measurements of DNA-bound TtgR for native (grey bars) and R176G mutant (white bars) in the presence of different effectors.

The 1 + 2 effector molecules per TtgR dimer stoichiometry is very unusual. To verify our interpretation, the binding of phloretin to a concentrated solution of TtgR was studied by isothermal titration calorimetry (ITC), which is illustrated in Figure 4. The binding curve is biphasic and a satisfactory curve fit was obtained with the “two independent sites” algorithm of the ORIGIN software (Microcal). An initial high affinity event (*K*_A_ = (2.1 ± 0.4) × 10^7^ *M*^− 1^, Δ*H* = − 15.1( ± 0.1) kcal/mol) can be distinguished from a second event characterized by a lower affinity (*K*_A_ = (4.6 ± 1.1) × 10^5^ *M*^− 1^) and a less favorable enthalpy change (Δ*H* = − 1.83( ± 0.13) kcal/mol). Most interestingly the *n* values associated with the first and second event were 1.02 ± 0.2 and 2.06 ± 0.06, respectively. This implies that one effector molecule binds with high affinity to the TtgR dimer (first event) followed by the binding of another two effector molecules to the dimer (second event). Therefore, the binding of the latter two phloretin molecules appears to occur in a unique thermodynamic event, which is further supported by the fact that the ligand is similarly positioned in both general binding sites in both monomers (Supplementary Data Figure 2(a) and (b)). In total, three phloretin molecules are necessary to saturate the TtgR dimer and data confirm our interpretation of the electron density.

### Mutagenesis studies to confirm the binding sites

Arg176 plays an important role in phloretin binding at the high affinity site. It also contributes to the one ligand molecule per TtgR dimer binding stoichiometry for the first binding event (Figures 3(f) and 4(a)). Interestingly, in *P. putida* KT2440 strain, which has a lower tolerance to toxic chemicals, this same position (residue 176) has a Gly instead of Arg (Figure 2). We therefore mutated Arg176 to Gly and investigated the binding of phloretin using ITC. The data can be fitted well with a one-site binding model with a 2:1 stoichiometry (two effector molecules per TtgR dimer) and the binding affinity is characterized by a *K*_A_ of (1.09 ± 0.06) × 10^5^ *M*^− 1^ (Table 1). These data are similar to that of the low affinity site (two effector molecules per TtgR dimer and *K*_A_ of (4.6 ± 1.1) × 10^5^ *M*^− 1^, Figure 4(a) and (b)). Overall, this observation is consistent with the interpretation that Arg176 mainly contributes to the high affinity site and one effector molecule per TtgR dimer stoichiometry of the high affinity site.

To verify that R176G indeed reduces the affinity of the high affinity site, we determined the crystal structure of TtgR^R176G^ in complex with phloretin. Initial 2*F*_o_–*F*_c_ electron density maps using TtgR without any ligands as a model show densities for phloretin in the lower affinity binding site (Supplementary Data Figure 2(c) and (d)), but not in the high affinity site. These data confirm that Arg176 contributes to the high affinity site, and the mutation of Arg176 to Gly significantly affects the high affinity site, while much less the lower affinity site. TtgR^R176G^ binds to naringenin similarly to wild-type TtgR (Table 1), confirming that Arg176 contributes mostly to the high affinity site.

To investigate the physiological effects of R176G mutant, electrophoretic mobility shift assays (EMSA) were used to measure the amount of DNA-bound TtgR after exposure to different effector molecules (cloramphenicol, naringenin and phloretin). These experiments show that TtgR^R176G^ does not release from its operator site in the presence of phloretin as efficiently as the wild-type TtgR (Figure 4(c)), whereas in the presence of chloramphenicol and naringenin, TtgR^R176G^ behaved similarly to the wild-type. Therefore, the mutation of R176 to G affects not only the binding of phloretin by TtgR but also its response to this flavonoid, since the release of TtgR^R176G^ from its operator site is reduced.

## Discussion

### Two distinct binding sites contribute to the versatile binding property of TtgR

Our work has identified two distinct binding sites within the large pocket in TtgR (Figure 1(c)), which contributes to the triangular shape of the binding pocket. The binding pocket is mainly hydrophobic, thus explaining its ability to bind to versatile aromatic ligands. The binding sites are not separated by the functionality of the compounds, being antibiotic or flavonoids, but by their chemical property, such as stereochemistry, conformation, and size. Phloretin, which has the highest binding affinity among the known ligands to TtgR,^13^ can bind to both sites with a total of three phloretin molecules observed within the TtgR dimer structure, one at the high affinity binding site, two at the low affinity binding sites. The phloretin in the high affinity binding site is located at the bottom of the binding pocket, lying horizontally, utilizing Arg176′ from the adjacent monomer to neutralize one of its hydroxyl groups. This in turn prevents the binding of phloretin to the high affinity site in the second monomer, explaining the 1:2 stoichiometry between phloretin and TtgR for this binding site. The high affinity site is smaller and significantly more buried (Figure 1(c)) and involves a number of specific interactions that contribute to the increased binding affinity (Figure 3(f)). Although naringenin has a similar size to phloretin, the hydrophobic environment provided by Val134 and Phe168 is not compatible with the charges of the naringenin chromenone ring.

In the case of quercetin, naringenin, tetracycline, chloramphenicol, as well as additional phloretin molecules, binding occurs vertically in the binding pocket with little specific interactions. This binding site is broader, more exposed, and contains less specific interactions (Figure 1(c)). We therefore term this site the general binding site, which most probably contributes to the versatility of TtgR's ligand binding property. However, these two sites are not completely separated. In fact, the bottom of the low affinity site overlaps with the high affinity site of phloretin, sharing several residues such as Leu66, His67, and Phe168.

R176G mutant TtgR binds to phloretin with reduced affinity and 1:1 stoichiometry (1 phloretin molecule per monomer), only to the low affinity site. Therefore, mutating this residue significantly affects the high affinity site and therefore the low affinity site becomes the dominant binding site. This is consistent with the observation that Arg176 mainly contributes to the high affinity site. However, TtgR^R176G^ binds to phloretin with a reduced affinity in the low affinity site, suggesting a potential positive cooperativity between the two sites in TtgR for phloretin binding. TtgR^R176G^ structure largely superposes well with wild-type TtgR structure. However, there are a number of side-chain conformational changes within the binding pocket, when TtgR and TtgR^R176G^ structures are compared, including Arg130, His67, and Phe168 (Supplementary Data Figure 2(e)). Residues 67 and 168 also contribute to the lower affinity site, providing possible explanations for the cooperative property of the two sites in phloretin binding.

### TtgR displays unique binding sites compared to other TetR family members

**Figure 5:**
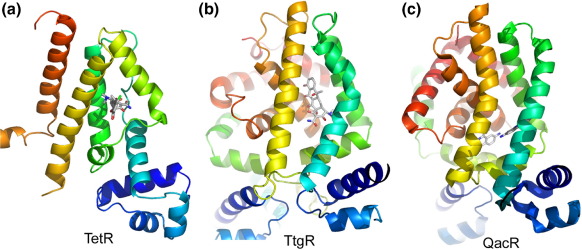
Comparison of TtgR with TetR and QacR ligand binding sites. (a) TetR binding to tetracycline (2TCT). Tetracycline is shown as sticks while TetR is shown as ribbons coloured from N to C termini. (b) Tetracycline binds to TtgR in a significantly different manner from TetR. (c) QacR binds to two drugs simultaneously (1QVU).

Structurally TtgR is a member of the TetR family of transcriptional repressors. The TetR protein family is characterized by a conserved HTH DNA binding domain and binds to DNA as a homodimer.^18^ However, the sequence conservation in the ligand binding domain is poor and the ligands that they recognize are diverse. TetR, the tetracycline repressor from which the TetR protein family derives its name, binds to tetracycline exclusively with high affinity (nM affinity). The tetracycline binding site in TetR is exclusively confined within one monomer and Mg^2+^ is chelated between hydroxyl groups of tetracycline and His110 of TetR.^19^ Tetracycline fits tightly into a well-defined binding pocket of TetR with its amino groups at the innermost point of the pocket and the cyclic ring on the outer side^19^ (Figure 5(a)). On the other hand, in TtgR, tetracycline binds vertically in the general binding site of TtgR, with its amide groups at the bottom of the pocket and the cyclic rings near the top of the pocket with little specific interactions, explaining its micromolar affinity to tetracycline (Figures 1(c), 3(b) and 5).

The TtgR structure displays strong similarity with that of QacR (1QVU), a multi-drug binding protein from *S. aureus*, in terms of secondary structures (Figure 5(b) and (c)) although the ternary structure is different (rmsd C^α^ 3.6 Å for the whole protein, rmsd C^α^ 0.9 Å for DNA binding domain, and 3.1 Å for ligand binding domain). QacR binds to a variety of positively charged ligands with micromolar affinities and a number of negatively charged residues within the pocket are responsible for this specificity.^20,21^ In TtgR, a general hydrophobic environment is provided in the binding pocket though a number of polar residues, including His114 and Asn110 are also involved in binding, explaining the presence of predominantly negative charges in TtgR ligands. In QacR, multiple binding sites are also identified though the binding pocket of TtgR is even broader than that of QacR (∼ 1500 Å^3^ compared to 1100 Å^3^).^21^ Furthermore, all the ligands bind to QacR with a 1:2 stoichiometry (one ligand per QacR dimer)^21^ while either 1:2 or 1:1 stoichiometry has been observed for TtgR. In QacR, the ligand entry portal is proposed to be near the dimer interface. Therefore binding to one monomer is proposed to obstruct the ligand entry to the adjacent monomer. However, in TtgR the most possible ligand entry is through the large portal formed at the bend of α4 (Figure 1(b)), well away from the dimer interface though some ligands utilize residues from the adjacent monomer.

### Implications in multidrug resistance

The data presented here explain how TtgR could recognize a wide range of antibiotics and plant secondary metabolites. Contrary to many other studies in multidrug resistance phenomena where many effectors characterized were laboratory reagents, the effectors studied here all have a physiological relevance. We identify two distinct binding sites within the protein. It is important to note that almost all the residues are conserved among most *Pseudomonas* species (Figure 2) including its close homologues *P. fluorescens* and *P. syringae*. The hydrophobic residues lining the side walls in the general binding site are also conserved among *E. coli*, *Neisseria meningitidis*, as well as *P. aeruginosa*, suggesting that the general binding site is conserved among these species.

Interestingly, *P. putida* DOT-T1E, one of the most resilient bacterial strains to toxic chemicals, has Arg instead of Gly or Tyr at position 176. Our structural data identify Arg176 as a crucial residue for the high affinity site. ITC data on TtgR^R176G^ protein confirm that changes at this residue result in reduced binding affinity to phloretin and possibly other effectors that utilize this binding site. EMSA results confirm that this reduced affinity of phloretin results in a reduced ability to release TtgR^R176G^ from bound DNA. These results highlight the importance of the regulatory system (not necessarily the efflux pumps themselves) in altering the ability of antibiotic resistance and open up avenues for developing effective antibiotics.

## Materials and Methods

### Protein expression, purification, and crystallisation

TtgR proteins were expressed and purified as described.^6,13,22^ In short, *ttgR* cloned into pET29a+ plasmid was over-expressed in *E. coli* B834(DE3) cells (Novagen) after induction with 1 mM IPTG, when *A*_600 nm_ reached 0.8. Proteins were expressed at 22 °C for 3 h. Cells were harvested by centrifugation and resuspended in 20 ml of Heparin A buffer (10 mM Tris-HCl (pH 6.4), 50 mM NaCl, 5% (v/v) glycerol. Cells were lysed by sonication and the suspension was centrifuged at 12,000***g*** for 30 min. The protein was then purified using a HiTrap Heparin HP column followed by gel filtration using a Superdex 200 HiLoad. The Se-Met TtgR was over-expressed in methionine auxotroph B834 cells grown in M9 minimal media supplemented with selenomethionine. Similar purification strategies were followed. Both TtgR and Se-Met TtgR were concentrated to 14 mg/ml. Crystals were grown using the sitting-drop vapor diffusion method in 0.2 M MgCl_2_, 0.1 M Bis-Tris (pH 6.5), 25% (v/v) PEG3350. The TtgR in complex with tetracycline was crystallized by mixing TtgR with tetracycline in 1:5 molar ratios and crystallized in 0.2 M MgCl_2_, 0.1 M Bis-Tris (pH 6.5), 10% PEG3350. TtgR in complex with chloramphenicol, phloretin, naringenin, and quercetin were crystallized using the same protocol and in similar conditions.

### Crystallographic data collection and structural determination

All crystals were soaked in crystallization buffer supplemented with 20% (v/v) glycerol as cryo-protectant before being frozen in liquid nitrogen. Datasets were collected under cryogenic conditions. All the datasets were collected at beamline ID29 at the European Synchrotron Radiation Facility or beamline 10.1 at the Daresbury Synchrotron Radiation Source. Data were processed using HKL2000 and scalepack^23^ or Mosflm.^24^ All the complexes crystallized in similar conditions and are in space group *P*2_1_2_1_2 with unit cell dimensions of 46, 231, and 44 Å. The structure of TtgR in complex with chloramphenicol was determined using selenomethionine substituted crystals and MAD methods^25^ using the selenium peak, inflection, and close remote energies. Five selenium sites were located and refined using SOLVE.^26^ Density modification and phase extension were carried out in RESOLVE.^26^ Subsequent model building/rebuilding were performed in the programs O^27^ and COOT.^28^ Model refinement was done using CNS^29^ by setting aside 10% of the observed reflection data for cross-validation. Subsequently, all the other structures were determined using the molecular replacement method implemented in the program PHASER^30^ and refined using CNS.^29^ All the structures were refined to 2.7–2.3 Å resolution. More than 88% residues are within the favoured regions of the Ramachandran plot and no residues are within disallowed regions. Clear electron densities were observed for most residues between positions 10 and 210 in all the structures except a number of loop regions (residues 75–81, 148–162) where weak electron densities were found, probably reflecting the flexibility and partial disorder of these regions, which contribute partially to the relatively high *R*_free_ values (Table 2). Significant extra electron densities were observed in a large pocket in the 2*F*_o_–*F*_c_ maps using TtgR without ligand as a model (Supplementary Data Figure 1). We attribute these densities with distinct shapes to the different ligand molecules bound. Solvent or small molecules in the crystallization buffers were ruled out due to the distinct shape and relatively large size of these densities. Ligand molecules were modeled in after many refinement cycles of TtgR alone guided by the crystallographic *R*_free_. In general, adding ligand molecules resulted in a 0.3–0.5% reduction in *R*_free_. The geometries of ligand molecules were adjusted in COOT using both real space fitting and manual adjustment to best fit into the electron density and satisfy chemical constraints. The final simulated annealing omit maps confirm the positioning of the ligand molecules (Supplementary Data Figure 1), and the quality of the density is in accord with the relatively low affinity of the ligands (*K*_D_ of ∼ 10–50 μM). The data quality and refinement statistics are summarized in Table 2.

### Isothermal titration calorimetry (ITC)

Measurements were performed on a VP-Microcalorimeter (MicroCal, Northampton, MA, USA) at 30 °C. The protein was thoroughly dialysed against 25 mM Pipes (pH 7.0), 250 mM NaCl, 5% (v/v) glycerol, 10 mM magnesium acetate, 10 mM KCl, 0.1 mM EDTA, 1 mM DTT buffer. The protein concentration was determined using the Bradford assay. Stock solutions of phloretin, chloramphenicol and naringenin at a concentration of 500 mM were prepared in dimethyl sulfoxide and the solutions were subsequently diluted with dialysis buffer to final concentrations of 1 mM (naringenin) or 2 mM (phloretin). The corresponding amount of dimethyl sulfoxide was added to the protein sample. Each titration involved injections of effectors into protein solution. Identical experimental conditions were used to analyse wild-type as well as mutant proteins. The mean enthalpies measured from injection of the ligands into the buffer were subtracted from raw titration data prior to data analysis with ORIGIN software (MicroCal).

### Electrophoretic mobility shift assay

Electrophoretic mobility shift assays were carried out as described.^6^ The DNA probe was a 189 bp fragment containing the *ttgABC-ttgR* intergenic region obtained from *P. putida* DOT-T1E chromosomal DNA by PCR. 1 nM of the radiolabelled probe (∼ 10,000 cpm) was incubated with 0.75 μM purified TtgR in 10 μl of DNA binding buffer (10 mM Tris-HCl (pH 7.0), 250 mM NaCl, 10 mM magnesium acetate, 10 mM KCl, 5% glycerol, 0.1 mM EDTA, 1 mM DTT) supplemented with 20 μg/ml poly(dI-dC) and 200 μg/ml bovine serum albumin. Effectors (prepared in DMSO) were added to the binding reaction at a final concentration of 1 mM. Reactions were incubated for 10 min at 30 °C and samples were run on 4.5% (w/v) non-denaturing polyacrylamide gels (Bio-Rad Mini-Protean II) for 2 h at 50 V at room temperature in Tris glycine buffer (25 mM Tris-HCl (pH 8.0), 200 mM glycine). The resulting gels were analysed using a Personal FX equipment and QuantityOne software (Bio-Rad).

### Structural analysis and molecular graphics

TtgR structures (full-length, DNA binding domain, and ligand binding domain) were submitted to DALI server^31^ to obtain structural homologues. Root-mean-square deviation (rmsd) of C^α^ atoms between TtgR and QacR were obtained from the DALI server. Distance calculations were performed using the CCP4 program LSQKAB.^32^ Cavity calculations were performed using CASTp.^33^ Sequence alignment and annotations were performed using ClustalW^34^ and Espript.^35^ All the Figures were produced using PyMol.^36^

### Protein Data Bank accession codes

All the structural data have been deposited to the RCSB Protein Data Bank through European Bioinformatics Institute and are available under accession codes 2UXP, 2UXO, 2UXI, 2UXU, 2UXH.

## Figures and Tables

**Table 1:** Thermodynamic parameters derived from the microcalorimetric titration of wild-type and mutant TtgR with different effector molecules

Effector	Proteins	*K*_A_ (M^− 1^)	*K*_D_ (μM)	Δ*G*(kcal/mol)	Δ*H*(kcal/mol)	*T*Δ*S*(kcal/mol)
Phloretin	TtgR^a^	(2.1 ± 0.4) × 10^7^	0.05 ± 0.01	− 10.2 ± 0.1	− 15.1 ± 0.1	− 4.9 ± 0.2
		(4.6 ± 1.1) × 10^5^	2.2 ± 0.5	− 7.8 ± 0.2	− 1.8 ± 0.2	6.0 ± 0.2
	TtgR^R176G^	(1.09 ± 0.06) × 10^5^	9.2 ± 0.5	− 7.0 ± 0.1	− 21.6 ± 0.5	− 14.6 ± 0.2
Naringenin	TtgR	(5.5 ± 0.1) × 10^4^	18 ± 0.3	− 6.6 ± 0.1	− 10.8 ± 0.1	− 4.2 ± 0.1
	TtgR^R176G^	(3.5 ± 0.3) × 10^4^	28.9 ± 2.5	− 6.3 ± 0.6	− 24.9 ± 3.5	− 18.6 ± 1.8

a Analysis with the “two binding-sites model” MicroCal version of ORIGIN. All the other data were analysed with the “one binding-site model”.

**Table 2:** Data collection and refinement statistics

						Semet		
	Phloretin	Quercetin	Naringenin	Tetracycline	Chloramphenicol	Peak	Inflection	Remote
*Data collection*								
Space group	*P*2_1_2_1_2	*P*2_1_2_1_2	*P*2_1_2_1_2	*P*2_1_2_1_2	*P*2_1_2_1_2		*P*2_1_2_1_2	
Cell dimensions								
*a*, *b*, *c* (Å)	47.2, 230.6, 43.9	46.7, 231.9, 44.3	46.6, 230.7, 44.0	46.2, 232.4, 44.0	46.5, 230.8, 44.0	46.7, 231.8, 44.2	46.7, 231.8, 44.2	46.7, 231.8, 44.2
α, β, γ (°)	90.0, 90.0, 90.0	90.0, 90.0, 90.0	90.0, 90.0, 90.0	90.0, 90.0, 90.0	90.0, 90.0, 90.0	90.0, 90.0, 90.0	90.0, 90.0, 90.0	90.0, 90.0, 90.0
Resolution (Å)	50–2.5	50–2.4	50–2.3	50–2.7	50–2.7	50–2.96	50–3.0	50–3.0
*R*_sym_ or *R*_merge_	6.5 (39.8)	9.4 (38.2)	5.4 (43.8)	9.6 (37.6)	9.9 (69.3)	7.0 (13)	5.6 (11)	6.4 (12)
*I*/s*I*	36.1 (6.2)	5.3 (1.5)	7.2 (1.7)	32.0 (7.9)	38.1 (5.3)	21.3(7.3)	23.5 (6.9)	22.5 (6.6)
Completeness (%)	92.9 (94.6)	96.6 (92.6)	90.5 (63.0)	92.9 (98.9)	96.2 (95.3)	93.43 (80.1)	94.86 (85.8)	94.6 (85.2)
Redundancy	6.6	5.0	5.7	5.3	8.6	5.9	5.7	5.6
Wavelength (Å)	0.9756	0.9756	0.9330	0.9756	0.9765	0.9802	0.9805	0.9763
Wilson *B*-factor (Å^2^)	56.1	37.4	43.7	44.5	46.1			

*Refinement*								
Resolution (Å)	50–2.5	50–2.4	50–2.3	50–2.7	50–2.7			
No. reflections	15,174	16,721	18,368	12,692	12,226			
*R*_work_/*R*_free_	24.5/29.4	23.7/29.5	24.3/29.1	23.1/29.4	23.2/29.6			
No. atoms	3432	3458	3283	3329	3322			
Protein	3207	3200	3122	3140	3169			
Ligand/ion	60	44	40	32	40			
Water	165	214	121	157	113			
*B*-factors (Å^2^)								
Overall	60.0	51.2	49.7	50.5	65.2			
Protein	61.2	51.3	49.1	49.8	66.2			
Ligand/ion	68.2	58.0	63.2	96.0	94.4			
Water	32.6	49.4	60.5	54.5	26.7			
r.m.s deviations								
Bond lengths (Å)	0015	0.014	0.0230	0.016	0.016			
Bond angles (°)	1.634	1.563	1.445	1.645	1.484			

*Ramachandran plot (%)*								
Favoured	91.0	91.0	89.2	89.7	91.0			
Allowed	9.0	9.0	10.5	10.3	9.0			
Generous	0.0	0.0	0.3	0.0	0.0			
Dissallowed	0.0	0.0	0.0	0.0	0.0			
